# Partial Oxidation of Methane to Syngas Over Nickel-Based Catalysts: Influence of Support Type, Addition of Rhodium, and Preparation Method

**DOI:** 10.3389/fchem.2019.00104

**Published:** 2019-03-13

**Authors:** Consuelo Alvarez-Galvan, Mayra Melian, Laura Ruiz-Matas, Jose Luis Eslava, Rufino M. Navarro, Mahdi Ahmadi, Beatriz Roldan Cuenya, Jose Luis G. Fierro

**Affiliations:** ^1^Structure and Reactivity Department, Instituto de Catálisis y Petroleoquímica, CSIC, Madrid, Spain; ^2^Department of Physics, University of Central Florida, Orlando, FL, United States; ^3^Department of Interface Science, Fritz Haber Institute of the Max Planck Society, Berlin, Germany

**Keywords:** syngas, methane, partial oxidation, nickel, rhodium, catalyst

## Abstract

There is great economic incentive in developing efficient catalysts to produce hydrogen or syngas by catalytic partial oxidation of methane (CPOM) since this is a much less energy-intensive reaction than the highly endothermic methane steam reforming reaction, which is the prominent reaction in industry. Herein, we report the catalytic behavior of nickel-based catalysts supported on different oxide substrates (Al_2_O_3_, CeO_2_, La_2_O_3_, MgO, and ZrO_2_) synthesized via wet impregnation and solid-state reaction. Furthermore, the impact of Rh doping was investigated. The catalysts have been characterized by X-ray diffraction, N_2_ adsorptiondesorption at −196°C, temperature-programmed reduction, X-ray photoelectron spectroscopy, O_2_-pulse chemisorption, transmission electron microscopy, and Raman spectroscopy. Supported Ni catalysts were found to be active for CPOM but can suffer from fast deactivation caused by the formation of carbon deposits as well as via the sintering of Ni nanoparticles (NPs). It has been found that the presence of Rh favors nickel reduction, which leads to an increase in the methane conversion and yield. For both synthesis methods, the catalysts supported on alumina and ceria show the best performance. This could be explained by the higher surface area of the Ni NPs on the alumina surface and presence of oxygen vacancies in the CeO_2_ lattice, which favor the proportion of oxygen adsorbed on defect sites. The catalysts supported on MgO suffer quick deactivation due to formation of a NiO/MgO solid solution, which is not reducible under the reaction conditions. The low level of carbon formation over the catalysts supported on La_2_O_3_ is ascribed to the very high dispersion of the nickel NPs and to the formation of lanthanum oxycarbonate, through which carbon deposits are gasified. The catalytic behavior for catalysts with ZrO_2_ as support depends on the synthesis method; however, in both cases, the catalysts undergo deactivation by carbon deposits.

## Introduction

The production of synthesis gas from methane is an important process for converting natural gas, one of the most abundant and cleanest-burning fossil fuels, into value-added high-quality liquid products (Gas-to-liquid technology, GTL). Steam reforming (SRM) has been the preferred technology for the industrial production of synthesis gas from methane to produce ammonia or methanol (Rostrup-Nielsen et al., 2002; Navarro et al., 2007a,b). Nevertheless, SRM is a highly energy-intensive process [Equation 1], and more energy-efficient alternatives to produce synthesis gas are sought.

(1) $${\text{CH}}_{4}+\;{\text{H}}_{2}\text{O}⇆\text{CO}+{3\text{H}}_{2}\;\Delta {\text{H}}_{298\text{K}}^{0}=+206\text{ kJ}/\text{mol}$$

Among the alternatives, the catalytic partial oxidation for the production of synthesis gas from methane (CPOM) is more energy efficient since it has fast kinetics and is exothermic, thus avoiding the need of large reactors and large amounts of superheated steam (Bharadwaj and Schmidt, 1995). In addition, the stoichiometry of the CPO [Equation 2] produces a synthesis gas with an H_2_/CO ratio of 2:1, which enables its direct utilization for methanol or Fischer-Tropsch synthesis without additional adjustment.

(2) $${\text{CH}}_{4}{\text{+ 1/2O}}_{2}⇆{\text{CO+2H}}_{2}$$

The catalytic partial oxidation of methane to syngas is challenging due to the difficulty in controlling the reaction selectivity toward total combustion. Several studies have been performed in the literature to describe the reaction mechanism involved in the CPOM and two reaction mechanisms have been proposed (Dissanayake et al., 1991; Hickman and Schmidt, 1992): the “direct mechanism,” in which CH_4_ and O_2_ react on the catalyst surface to yield CO and H_2_, and the “combustion-reforming mechanism,” in which CH_4_ and O_2_ react first to form H_2_O and CO_2_ and then, with the excess methane, the dry reforming of CO_2_ [Equation 3] and steam reforming [Eq.4] produce the final CO and H_2_.

(3) $${\text{CH}}_{4}\text{+ }{\text{CO}}_{2}⇆\text{2CO + }{\text{2H}}_{2}$$

(4) $${\text{CH}}_{4}\text{+ }{\text{H}}_{2}\text{O}⇆\text{CO + }{\text{3H}}_{2}\;$$

Due to the excessive temperature gradients at high conversion rate, the exothermic nature of the reaction and fast deactivation due to carbon deposition on the catalyst's surface, the development of efficient catalysts for the CPOM has been challenging. NPs of noble metals [Pt (Ji et al., 2001), Rh (Ruckenstein and Wang, 1999; Puolakka and Krause, 2007), Ru (Ashcroft et al., 1990), Pd (Vernon et al., 1990)] and non-noble metals [mainly Ni and Co (Wang and Ruckenstein, 2001)] supported on various oxide substrates have been studied in the CPOM reaction. Nickel is one of the most widely used active phases for CPOM (Miao et al., 1997; Ostrowski et al., 1998; Takehira et al., 2004; Wang et al., 2004). As compared to noble metals, nickel is inexpensive but suffers from deactivation on-stream as a consequence of several processes such as sintering, carbon deposition (Claridge et al., 1993) or solid-state reactions of nickel with the substrate. From the large body of work developed on the CPOM reaction, it is clear that the activity and stability of nickel catalysts depend on both the active phase and the support. Metal particle size was proven to be an important factor for the initial intrinsic activity and for the rate of deactivation, with both decreasing with increasing active metal particle sizes (Barbier and Marecot, 1986; Barbier, 1987). The influence of the support on the performance of Ni-based catalysts has been widely studied in the literature (Tsipouriari et al., 1998). Non-reducible Al_2_O_3_ is one of the most studied oxides as support for Ni catalysts because of its thermal stability and high ability to disperse Ni nanoparticles (NPs) (Hu and Ruckenstein, 1998; Ostrowski et al., 1998; Zhang et al., 2000); however, its application for CPOM reaction is limited because of the relatively high deactivation of Ni NPs by sintering and the formation of coke deposits (Lu et al., 1998). Magnesium oxide is another non-reducible support widely studied to disperse stable Ni particles (Choudhary et al., 1998a; Ruckenstein and Hu, 1999; Nishimoto et al., 2004). In this case the formation of a solid solution between nickel and magnesia (Mg_1−x_Ni_x_O) only allows for the reduction of a small fraction of the nickel that remains in close interaction with the basic MgO substrate, favoring this structure for the production of syngas by CPOM with high activity (Requies et al., 2005). Lanthanum oxide has also been used as a support for Ni catalysts (Tsipouriari et al., 1998; Nishimoto et al., 2004). For the Ni/La_2_O_3_ catalysts, good stability was reported and attributed to the increased metal-support interface because the nickel NPs are decorated by La_2_O_2_CO_3_ species that promote the gasification of coke. Reducible supports (CeO_2_, ZrO_2_) have been also studied as systems to disperse active and stable nickel particles for CPOM. CeO_2_ is known for its ability to improve the dispersion and stabilization of small nickel metal NPs and for its high oxygen storage/transport capacity, which allows for continuous removal of carbonaceous deposits from active sites (Choudhary et al., 1993; Diskin et al., 1998). In addition, under reducing conditions, the SMSI (*Strong Metal-Support Interaction*) effect could be observed on ceria, which in turn affects the stability and activity of the dispersed nickel particles (Trovarelli, 1996). Zirconia is another support that shows interesting properties for the dispersion of active and stable Ni NPs. However, the application of ZrO_2_ for CPOM reaction is debatable, since this support decreases the availability of the oxygen that participates in the direct CPOM to synthesis gas, resulting in a decrease in activity (Pompeo et al., 2005).

The incorporation of a second metal to the Ni-based catalysts is a common practice designed to improve catalyst stability. The beneficial effect of adding small amounts of precious metals such as Ru, Pt, Pd, Ir, and Rh to a Ni catalyst was previously demonstrated (Tomishige et al., 2002). Rh is one of the most promising metals (Tanaka et al., 2010a). The improvement was explained in terms of the H-spillover from the noble metal towards the non-noble metal, helping the non-noble metal surface to stay metallic (Chen et al., 1997).

As stated above, efficient Ni catalysts for the CPOM reaction require control over the electronic and structural properties of the nickel NPs, which could be achieved by the careful selection of the support and synthesis procedure. In this context, the main objective of this work is to study the influence of different supports (Al_2_O_3_, CeO_2_, La_2_O_3_, MgO, ZrO_2_) with different textural and surface (basicity, reducibility) properties on the activity, selectivity and stability of Ni-based catalysts for the CPOM reaction at atmospheric pressure. Two different synthesis methods have been used: (I) impregnation over different commercial supports and (II) solid state reaction. The influence of a small amount of Rh over the reducibility and reactivity of the catalysts has been tackled. Using various characterization techniques, we will establish structure-activity relationships that indicate which catalyst properties determine its reactivity and thus give us a way to improve the catalytic performance of the systems.

The Ni catalysts have been characterized by X-ray diffraction, nitrogen adsorption-desorption, temperature programmed reduction, X-ray photoelectron spectroscopy, oxygen-pulse chemisorption, transmission electron microscopy, and Raman spectroscopy. The evolution of the structure and morphology of the catalysts is reported and correlated to its catalytic performance.

## Materials and Methods

### Preparation of Catalysts

#### Catalysts Prepared by Impregnation of Commercial Supports

Ni (5% weight) catalysts supported on commercial Al_2_O_3_ (Johnson Matthey, 99.97%), CeO_2_ (Johnson Matthey, 96%), La_2_O_3_ (Fluka Chemika, 99.98%), MgO (Fluka Chemika, >98%) and ZrO_2_ (Johnson Matthey, 99.5%) were prepared by wet impregnation. First, the supports were thermally stabilized by calcination at 850°C over 3 h. Then, they were sieved between 212 and 425 μm. The corresponding amount of Ni(NO_3_)_2_·6 H_2_O (Aldrich Chemie) was dissolved in distilled water. Each support was impregnated with this solution by rotary evaporation (70°C, 1 h). Then, the obtained solid was dried at 110°C for 2 h and calcined at 500°C for 3 h.

#### Catalysts Prepared by Solid State Reaction

The RhNi (0.1% Rh, 10% Ni, weight) catalysts, supported on Al_2_O_3_, CeO_2_, La_2_O_3_, MgO, and ZrO_2_, were prepared by mixing and grounding in an agate mortar the corresponding amounts of Ni(NO_3_)_2_·6H_2_O (assay > 98.5%, Aldrich) and the nitrates of the support cations (Mg(NO_3_)_2_·6H_2_O (Scharlau, 98%), La(NO_3_)_2_·6H_2_O (Johnson Matthey, 99.9%), Al(NO_3_)_3_·9H_2_O (99.997% Sigma Aldrich), Ce(NO_3_)_3_·6H_2_O (Johnson Matthey, 99.5%), and ZrO(NO_3_)_2_·xH_2_O, 99.9%), respectively. Since the Rh amount is very small, the method to incorporate this element was based on dissolving the corresponding amount of dicarbonyl(acetylacetonate) Rh(I) (Rh(CO)_2_acac) (Sigma Aldrich, purum) in acetone and adding this solution dropwise to the powder mixture of nickel nitrate and the corresponding support cation nitrate, thoroughly removing it with a spatula. Then, each mixture was dried at 110°C for 2 h and calcined at 500°C for 3 h. Finally, the samples were ground and sieved between 212 and 425 μm.

### Characterization Techniques

#### X-Ray Diffraction

XRD patterns were recorded on a Seifert 3000 powder diffractometer using Cu Kα radiation (λ = 0.15418 nm) generated at 40 kV and 40 mA. Scans were recorded at a rate of 0.02°/s for 2θ diffraction angles between 10 and 90°.

#### N_2_ Adsorption-Desorption Isotherms

Textural properties were evaluated by N_2_ adsorption-desorption isotherms of the samples recorded at liquid nitrogen temperature with a Micromeritics ASAP2000 apparatus. Samples were degassed at 150°C under vacuum overnight. Specific areas were calculated by applying the BET method.

#### Temperature Programmed Reduction

Temperature-programmed reduction (TPR) experiments were carried out using a semiautomatic Micromeritics TPD/TPR 2900 apparatus equipped with a TC detector. Prior to the reduction experiments, the samples (ca. 30 mg) were thermally treated under air stream at 300°C to remove moisture. TPR profiles were obtained by heating the samples under a 10% H_2_/Ar flow (50 mL/min) from 25 to 800°C with a linear rate of 10°C/min.

#### Oxygen Chemisorption Capacity

Oxygen chemisorption capacity was determined by O_2_-pulse chemisorption. First, the catalyst sample (60–70 mg) was inserted in a U-quartz reactor and heated to 500°C in argon, maintaining this temperature for 15 minutes. Then, the temperature was decreased to room temperature and the sample was reduced under a H_2_/Ar flow (10% H_2_) up to 750°C over 60 minutes. Finally, the carrier gas was changed to helium, and once the baseline was stabilized, O_2_ pulses were injected until the O_2_ detected peaks showed the same area.

#### X-Ray Photoelectron Spectroscopy

To extract information about the chemical state and composition of the Ni-supported samples, XPS measurements were acquired using a monochromatic X-ray source (Al Kα, 1486.6 eV) operating at 200 W and a hemispherical electron analyzer (Phoibos 100, SPECS GmbH). The high-resolution data were acquired with a pass energy of 18 eV. CasaXPS software was used to analyze the data.

X-ray photoelectron spectra of the RhNi supported samples were recorded on a VG Escalab 200R spectrometer equipped with a hemispherical electron analyzer and Mg Kα (h·ν = 1253.6 eV) X-ray source (12 kV and 10 mA). The powder samples were degassed at 150°C for 1 h before being transferred into the analysis chamber. Charge effects on the samples were corrected by fixing the binding energies of the C1s peak at 284.9 eV due to adventitious carbon. This reference gave binding energy values with an accuracy of ±0.1 eV. The data were treated with the “XPS peak” software. The spectra were decomposed with the least squares fitting routine using Gaussian/Lorentzian functions after subtracting the Shirley background.

#### Raman Spectroscopy

Raman spectra of the samples were recorded in air, under ambient conditions (being the samples hydrated by air humidity), using a single monochromator Renishaw inVia 1000 system equipped with a thermoelectrically cooled CCD detector and holographic super-Notch filter. The samples were excited with 535 nm (1800 lines/mm).

### Catalytic Activity Tests

The catalytic behavior of the different catalyst precursors for the partial oxidation of methane to syngas was studied under atmospheric pressure at 750°C using a stainless-steel fixed bed reactor (length = 150 mm, internal diameter = 9 mm) placed inside a hinged oven. The catalysts (100 mg) were subjected to pretreatment under 50 mL_N_/min of H_2_/N_2_ (10% H_2_, molar) for the Ni-supported samples or under N_2_ flow at 750°C for 1 h for the RhNi-supported samples, prior to the reaction. Then, the reactants were fed to the reactor (22.6 mL_N_/min N_2_, 6 mL_N_/min O_2_, and 12 mL_N_/min CH_4_). The reaction stream was analyzed on-line by gas chromatography (Varian 45-GC) with a thermal conductivity detector, equipped with a 5A molecular sieve (CP7538) to separate H_2_, N_2_, CH_4_ y CO and a PoraBOND Q (CP7354) column to separate CO_2_ and H_2_O with a thermal conductivity detector. Argon was used as a carrier gas in order to increase the sensitivity for H_2_ detection.

Methane conversion, CO selectivity, H_2_ yield and H_2_/CO ratio were defined as follows:

$$\begin{array}{l} \;CH_{4}conversion\;\left(\%\right)\\ =\;\frac{CH_{4}\;molar\;flow\;\left(inlet\right)-CH_{4}\;molar\;flow\;(outlet)}{CH_{4}\;molar\;flow\;(inlet)}\;\cdot \;100\\ \;H_{2}\;yield\;\left(\%\right)=\;\frac{\;H_{2}\;molar\;flow\;\left(oulet\right)}{2\cdot CH_{4}\;molar\;flow\;(inlet)}\;\cdot \;100\\ \;\;CO\;selectivity\;\left(\%\right)\\ =\;\frac{CO\;molar\;flow\;\left(oulet\right)}{CH_{4}\;molar\;flow\;\left(inlet\right)-CH_{4}\;molar\;flow\;(outlet)}\;\cdot \;100\\ \;\;\frac{H_{2}}{CO}(molar\;ratio)=\;\frac{H_{2}molar\;flow\;\left(oulet\right)}{CO\;molar\;flow\;(outlet)} \end{array}$$

## Results and Discussion

The catalysts have been analyzed before reaction (section Physicochemical Characterization of Calcined and Reduced Samples) by XRD, adsorption-desorption of N_2_ at −196°C, TPR, and XPS and after reaction (section Physicochemical Characterization of Used Catalysts) by XRD, Raman spectroscopy and XPS. Finally, the results from the activity tests are presented and discussed (section Activity Tests).

### Physicochemical Characterization of Calcined and Reduced Samples

#### Structural Properties

**Figure 1:**
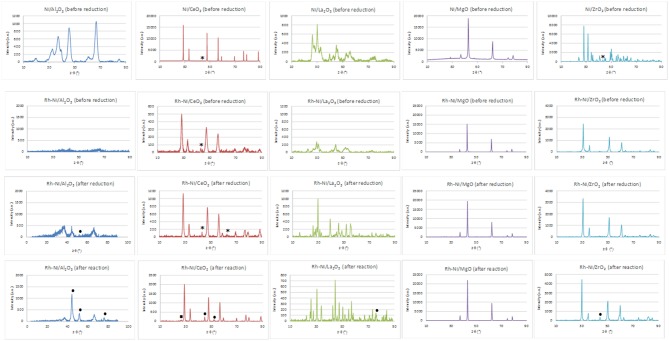
XRD of Ni (prepared by impregnation) and RhNi samples (prepared by solid state reaction) (NiO (01-073-1519): *; Ni^0^ (00-001-1258): • ; CeO_2−x_ (00-049-1415): □).

X-ray diffraction patterns of nickel-supported catalysts prepared by the wetness impregnation method and acquired after calcination (before reduction) are depicted in the first line of Figure 1. These diagrams show the diffraction lines corresponding to each support (Al_2_O_3_: 00-048-0367; CeO_2_, cubic phase: 01-075-0076; La_2_O_3_, hexagonal: 01-074-2430; MgO, cubic phase: 01-075-0447 and monoclinic ZrO_2_: 01-078-1807). The crystallinity of the supports is very different, being higher for the MgO-supported catalyst. Only for the ceria- and zirconia-supported catalysts is it possible to distinguish the main diffraction line related to NiO crystallites (cubic phase (01-073-1519), corresponding to the (200) diffraction plane. By applying the Scherrer equation, an average domain of NiO crystallites was measured (Table 1). On the other hand, the crystalline domains differ among the different supports, although being smaller and quite similar for the alumina and lanthana-supported catalysts.

**Table 1:** Average domain sizes for calcined samples (before reduction) prepared by impregnation (determined by the Scherrer equation).

**Sample**	**Phase**	**hkl**	**2·θ (^**°**^)**	***t* (nm)**	**Phase**	**hkl**	**2·θ (^**°**^)**	***t* (nm)**
Ni/Al_2_O_3_	Al_2_O_3_	4-4-2	66.867	5.0				
Ni/CeO_2_	CeO_2_	1-1-1	28.743	58.3	NiO	2-0-0	43.466	19.6
Ni/La_2_O_3_	La_2_O_3_	1-0-0	26.366	5.5				
Ni/MgO	MgO	2-0-0	42.782	15.1				
Ni/ZrO_2_	ZrO_2_	1-1-1	28.115	29.2	NiO	2-0-0	43.218	18.9

For the Ni/MgO and Ni/Al_2_O_3_ catalysts, the peaks corresponding to nickel oxide overlap with those corresponding to the respective supports. SimilarQ16ly, in the Ni/La_2_O_3_ catalyst, the incorporated NiO reacts on the La_2_O_3_ surface to form a LaNiO_3_ phase (00-012-0751), whose peaks overlap with those corresponding to La_2_O_3_. On the other hand, for the Ni-MgO sample, the formation of a NiO-MgO solid solution cannot be discarded since their diffraction lines coincide with those of MgO.

**Figure 2:**
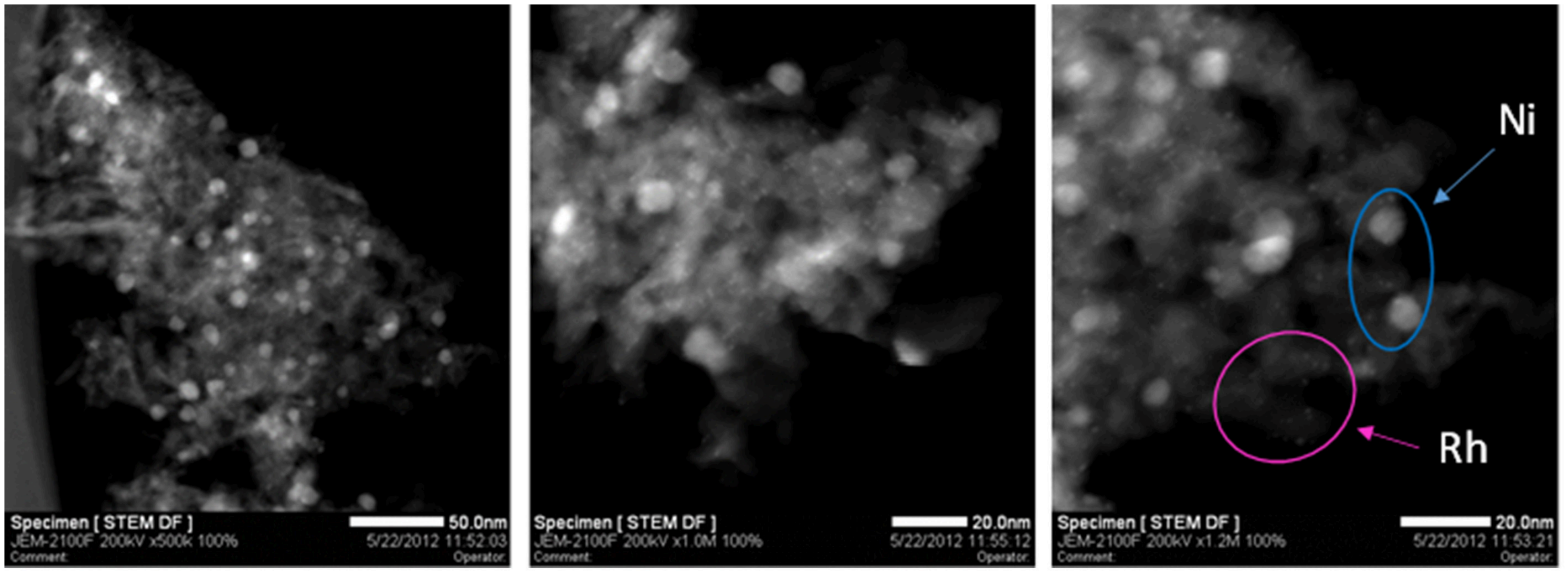
STEM micrographs of sample Rh-Ni/Al_2_O_3_ prepared by impregnation.

The Rh-Ni/Al_2_O_3_ catalyst prepared by impregnation shows a diffractogram similar to that of the Ni/Al_2_O_3_ catalyst (not displayed). In order to determine the particle size of nickel and rhodium particles, the Rh-Ni/Al_2_O_3_ catalyst was reduced according to the same activation procedure used for the activity tests and studied by STEM, Figure 2. Rh NPs appear as finely dispersed particles with an average size less than 1 nm. Ni NPs are also observed and present larger size (average around 8 nm).

In addition, the XRD patterns of Rh-promoted Ni-supported catalysts, prepared by solid state reaction, were recorded. The XRD patterns of the calcined ones (before reduction) are shown in Figure 1 (line 2). As for the catalysts prepared by impregnation, the diffraction lines are mainly ascribed to the corresponding supports. In the case of the lanthana-supported catalyst, the formation of the LaNiO_3_ phase cannot be discarded. For the catalyst supported on magnesia, the diffraction lines corresponding to MgO and a Ni-Mg-O solid solution cannot be distinguished because of their strong overlap (Arena et al., 1996). The formation of the Ni-Mg-O solid solution is due to the relatively high calcination temperature used in the catalyst preparation. Concerning the zirconia-supported catalyst, for this preparation method, zirconia crystallizes in the tetragonal phase.

Rh reflections were not observed, as expected from the very small crystallite size. Concerning the presence of crystalline nickel species, only in the case of the catalyst supported on CeO_2_ was it possible to observe a small diffraction line of the (2 0 0) plane of NiO. The crystallinity of the catalysts considerably changed; being higher for the MgO-supported catalyst and lower for the Al_2_O_3_-supported one. The XRD diagram of this last catalyst indicates its amorphous nature, since the calcination temperature was not high enough to get a crystalline structure of alumina. The temperature range needed to form the gamma phase of alumina is 600–875°C (Sathyaseelan et al., 2013).

The Scherrer equation was applied to these X-ray diffraction diagrams, and the average domain sizes of the different crystalline phases are shown in Table 2. For these calcined samples, prepared by solid-state reaction, the average domain size among the different supports did not change as much as that which occurred for the calcined samples prepared by impregnation of commercial supports. Moreover, it is noteworthy that the crystalline domains of NiO, if they could be observed, were smaller than those obtained for the samples prepared by impregnation of the commercial supports (Table 1).

**Table 2:** Average domain sizes for calcined samples (before reduction), fresh reduced catalysts and catalysts after reaction prepared by solid state reaction.

**Sample**	**Phase**	**hkl**	**2·θ (^**°**^)**	***t* (nm)**	**Phase**	**hkl**	**2·θ (^**°**^)**	***t* (nm)**
**CALCINED SAMPLES (BEFORE REDUCTION)**								
Rh-Ni/CeO_2_	CeO_2_	1-1-1	28.592	8.2	NiO	2-0-0	43.242	12.5
Rh-Ni/La_2_O_3_	La_2_O_3_ cubic	4-0-0	30.733	6.4	La_2_O_3_ hexag.	0-1-1	29.381	8.6
Rh-Ni/MgO	MgO	2-0-0	42.878	29.6				
Rh-Ni/ZrO_2_	ZrO_2_	1-1-1	30.575	17.4				
**FRESH REDUCED CATALYSTS**								
Rh-Ni/Al_2_O_3_					Ni^0^	2-0-0	52.041	13.5
Rh-Ni/CeO_2_	CeO_2_	1-1-1	28.548	11.3	NiO	0-1-2	43.261	19.4
Rh-Ni/La_2_O_3_	La_2_O_3_ hexag.	0-1-1	29.8968	20.5	Ni^0^	1-1-1	44.440	21.0
Rh-Ni/MgO	MgO	2-0-0	42.8532	22.0				
Rh-Ni/ZrO_2_	ZrO_2_	1-1-1	30.4207	15.8				
**CATALYSTS AFTER REACTION**								
Rh-Ni/Al_2_O_3_					Ni^0^	2-0-0	51.764	7.4
Rh-Ni/CeO_2_	CeO_2_	1-1-1	28.804	17.2	Ni^0^	1-1-1	44.7199	21.3
Rh-Ni/La_2_O_3_	La_2_O_2_CO_3_	1-0-3	30.614	21.4	Ni^0^	2-2-0	75.8017	12.8
Rh-Ni/MgO	MgO	2-0-0	42.889	26.4				
Rh-Ni/ZrO_2_	ZrO_2_	1-0-1	30.218	20.7	Ni^0^	1-1-1	44.3868	19.2

This figure also shows the XRD patterns of the reduced catalysts (Figure 1, line 3). The diffraction lines correspond basically to that of the supports. In the case of the lanthana-supported catalyst, the XRD profile consists of overlapping lines of La_2_O_3_, La_2_NiO_4_ (tetragonal, 01-079-0953) and La(OH)_3_ (hexagonal, 01-083-2034) as a result of the strong interaction between nickel and lanthana (Pantaleo et al., 2015). Only for the catalyst supported on alumina and lanthana, low intensity peaks assigned to Ni metal [at 51.8°, plane (200)] can be distinguished. On the other hand, diffraction lines assigned to NiO are observed in the ceria-supported samples, indicating that at least part of the nickel phase cannot be reduced.

#### Textural Properties

**Figure 3:**
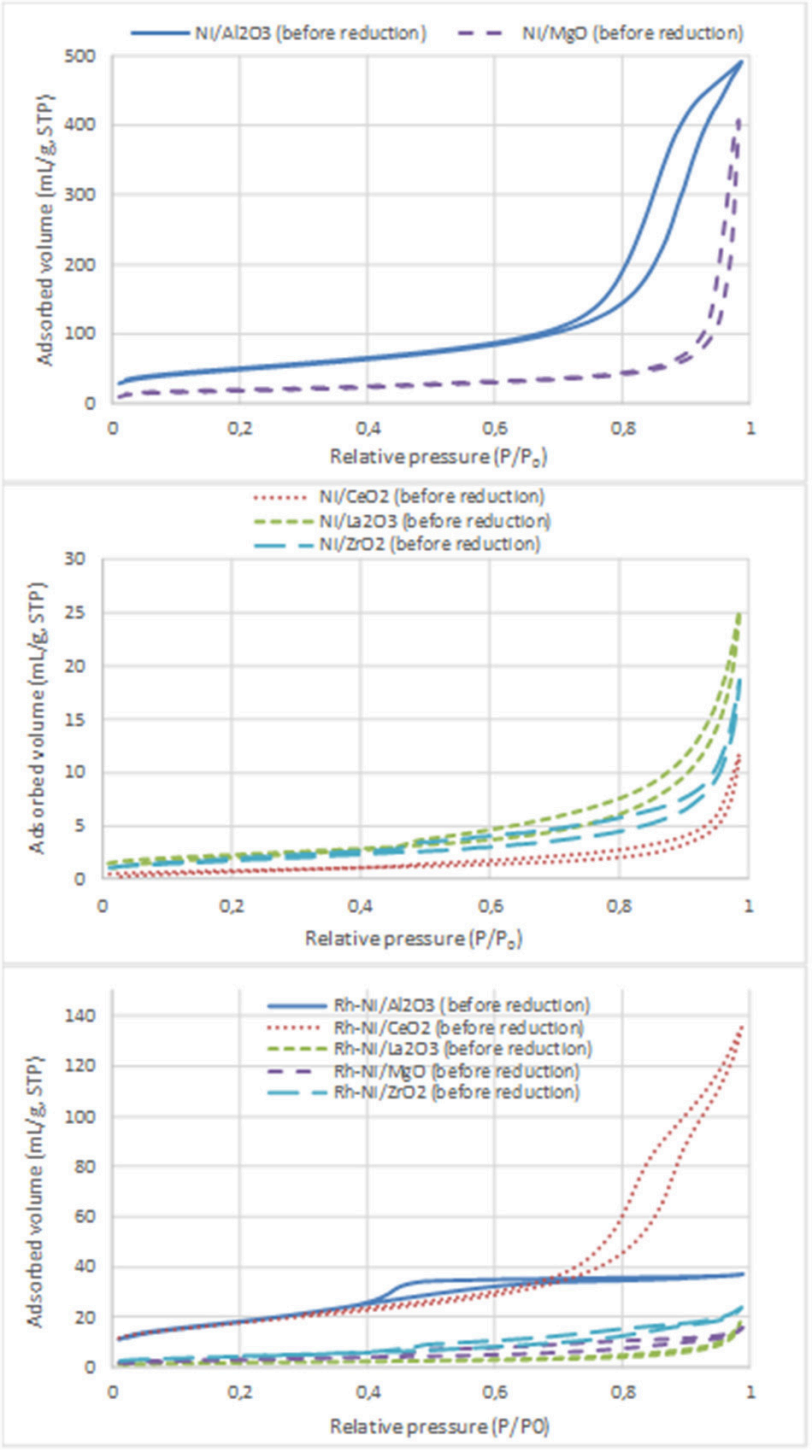
N_2_ adsorption-desorption isotherms of the calcined samples (Ni catalysts, prepared by impregnation and Rh-Ni catalysts prepared by solid-state reaction).

Nitrogen adsorption-desorption isotherms of Ni- and Rh-Ni-supported catalysts are displayed in Figure 3 and the surface areas are summarized in Table 3. These isotherms, which are of type IV, are assigned to mesoporous materials. All isotherms display a type H3 hysteresis loop, indicating that the catalysts contain a mesoporous network consisting of slit-type pores. In the relative pressure range of 0.7–1, the loop originates from larger and usually disordered interparticle pores (Sing et al., 1985).

**Table 3:** BET surface areas of commercial supports, of calcined Ni samples prepared by impregnation, and Rh-Ni samples prepared by solid state reaction (calcined, reduced and after reaction).

**BET surface areas of supports and catalysts (m^2^/g)**				
**COMMERCIAL SUPPORTS**				
Al_2_O_3_	CeO_2_	La_2_O_3_	MgO	ZrO_2_
182.5	1.2	1.7	164.6	5.4
**CATALYSTS PREPARED BY IMPREGNATION OF COMMERCIAL**				
**SUPPORTS**				
Ni/Al_2_O_3_	Ni/CeO_2_	Ni/La_2_O_3_	Ni/MgO	Ni/ZrO_2_
**(Calcined samples)**				
176.9	3.1	8.2	60.5	6.5
**CATALYSTS PREPARED BY SOLID STATE REACTION**				
Rh-Ni/Al_2_O_3_	Rh-Ni/CeO_2_	Rh-Ni/La_2_O_3_	Rh-Ni/MgO	Rh-Ni/ZrO_2_
**(Calcined samples)**				
66.6	63.8	6.1	10.9	16.0
**(Fresh reduced catalysts)**				
37.9	43.0	12.5	15.4	8.4
**(Catalysts after reaction)**				
38.5	23.6	5.5	5.7	9.6

For the Ni catalysts prepared by impregnation, it can be seen that the adsorbed amount of N_2_ is higher for the catalysts supported on alumina and magnesia and smaller for the ceria- and zirconia-supported ones. This is in accordance with the large crystallite size of these commercial supports and their low surface area (see Table 3), which in turn decreases the dispersion of the nickel phase, as can be derived from the X-ray diffraction diagrams (Figure 1) and the average crystalline domains determined for supported NiO NPs (Table 1).

For the catalysts supported on alumina and magnesia, a decrease in surface area is observed, which is explained by the blockage of part of the porous lattice by nickel oxide NPs. On the contrary, for the samples supported on ceria, lanthana, and zirconia, an increase in surface area is observed, which might be attributed to the contribution of these NiO surface NPs. The catalyst with the largest surface area is, by far, the one supported on alumina, followed by that supported on magnesia. The other catalysts show BET surface area values one order of magnitude lower, being the lowest that supported on ceria.

On the contrary, the Rh-Ni calcined catalyst, prepared by solid state reaction and supported on ceria, presents larger porosity as derived from the higher extent of nitrogen adsorption and the larger hystheresis loop shifted to high relative P/P_0_ pressure, indicating the formation of larger pores.

The alumina-supported Rh-Ni calcined catalyst prepared by solid state reaction, presents a H_2_ hysteresis loop in the range of small mesopores at relative pressures of 0.4–0.6, indicating that the catalysts contain complex mesoporous networks consisting of pores with ill-defined shapes. From the nitrogen adsorption values, it can be seen that this preparation method gives catalysts with more uniform textural properties than those prepared by impregnation of commercial supports. This is reflected in the surface areas that are between 6 and 39 m^2^/g for the samples used in the reaction, the trend being as follows: Rh-Ni/Al_2_O_3_ > Rh-Ni/CeO_2_ > Rh-Ni/ZrO_2_ > Rh-Ni/MgO ~ Rh-Ni/La_2_O_3_. The highest surface area was obtained for the ceria-supported catalyst as a consequence of the formation of small nanocrystals (Kundakovic and Flytzani-Stephanopoulos, 1998).

The reduction of these Rh-Ni catalysts produced a change in surface area with respect to the corresponding calcined counterparts, increasing for lanthana and magnesia-supported catalysts and decreasing for the other ones. The used catalysts showed a decrease in surface area due to sintering, with the exception of those supported on alumina and zirconia, for which the surface area slightly increased.

#### Redox Properties

**Figure 4:**
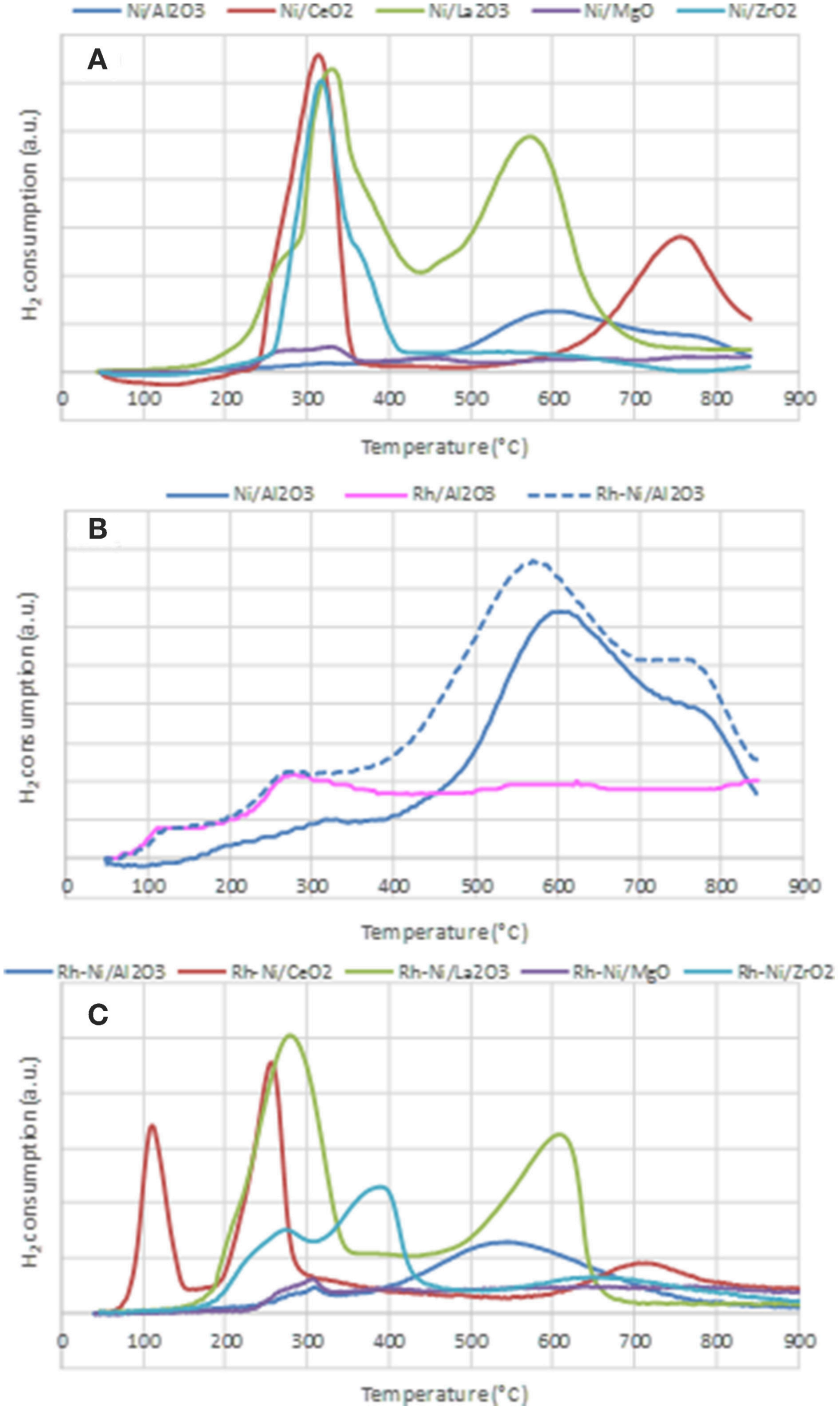
Temperature Programmed Reduction profiles of the calcined samples, prepared by impregnation **(A,B)** and prepared by solid state reaction **(C)**.

The reduction profiles of the different catalysts prepared by impregnation and by solid state reaction are shown in Figure 4. The reduction profiles of Ni catalysts prepared by impregnation are depicted in Figure 4A. The H_2_ consumption profile corresponding to the alumina-supported sample shows a very small contribution around 300°C due to the reduction of free NiO particles and wide consumption between 400 and 850°C, which indicates the formation of nickel oxide particles with different interactions with the support. The consumption at temperatures higher than 700°C is related to the reduction of nickel aluminate (Poncelet et al., 2005).

For the calcined Ni/CeO_2_ sample, two peaks are observed: the first one is centered around 320°C and is ascribed to the reduction of free NiO NPs, while the second and wider one, centered around 780°C, is associated with the reduction of surface ceria and NiO NPs strongly interacting with the support. The consumption at the highest recorded temperature is explained by the reduction of some bulk ceria (Zhang et al., 2008).

The reduction profile of Ni-La_2_O_3_ calcined sample is characterized by two consumption peaks, the first one centered around 330°C and the second one around 580°C, both due to the 2-step reduction of the perovskite LaNiO_3_ (see Equations 5, 6), formed during the calcination step (Barbero et al., 2003).

(5) $$LaNiO_{3}+1/2H_{2}\to \;LaNiO_{2.5}+1/2H_{2}O$$

(6) $$LaNiO_{2.5}+H_{2}\to \;1/2La_{2}O_{3}+Ni+H_{2}O$$

In the first step at lower temperature, Ni^3+^ species stabilized in the perovskite structure are reduced to Ni^2+^, and the resulting compound, LaNiO_2.5_, is an oxygen-deficient structure. In the second reduction step, a complete reduction of nickel to metallic nickel is achieved, and a system based on finely dispersed Ni^0^ particles supported on a La_2_O_3_ matrix is obtained.

The reduction profile of the Ni/MgO calcined sample is characterized by very low H_2_ consumption, indicative of an extensive formation of a Ni-Mg-O solid solution (Kirillov et al., 2011), which is difficult to reduce under the conditions used in TPR (Temperature Programmed Reduction) analysis. The peak around 330°C is related to the reduction of free NiO particles. The consumption between 400 and 800°C is assigned to the reduction of Ni^2+^ species in the outer or subsurface layers of the MgO lattice. For higher temperatures, H_2_ consumption might be due to the reduction of Ni^2+^ taking place in the Ni-Mg-O solid solution (Wang et al., 2009a). For Ni/ZrO_2_, a main reduction peak centered at 320°C, with a shoulder around 350°C can be found. The main H_2_ consumption is due to the reduction of free NiO NPs and the minor one, at some higher temperature, to the reduction of NiO strongly interacting with the support (Wang et al., 2009b).

Figure 4B depicts the reduction profiles of the calcined samples Ni/Al_2_O_3_, Rh/Al_2_O_3_, and Rh-Ni/Al_2_O_3_, prepared by impregnation of commercial alumina. The reduction profile of Ni/Al_2_O_3_ has already been described above. The reduction profile of the Rh/Al_2_O_3_ sample shows two small contributions ascribed to the reduction of Rh_2_O_3_ to Rh^0^. As reported in the literature, it is expected that calcination at 500°C causes a fraction of the Rh phase to interact strongly with alumina, with Rh becoming incorporated into the surface layer of this support and, thus, being difficult to reduce. Therefore, the peak around 125°C is attributed to the reduction of rhodium oxide particles containing low interaction with the support, and the second one, around 280°C, is due to the reduction of rhodium oxide species with strong interaction with alumina (Burch et al., 1996). The profile of the Rh-Ni/sample reflects the reduction of both Rh oxide and Ni oxide species. However, a shift to lower temperatures as compared to that of Ni/Al_2_O_3_ can clearly be observed. This is because the presence of noble metals facilitates the reduction of nickel oxide via a hydrogen-spillover mechanism (Tanaka et al., 2010b; Berger-Karin et al., 2011).

Figure 4C displays the TPR profiles of the Rh-Ni calcined samples prepared by solid-state reaction. The profile of the sample supported on alumina shows two contributions, a minor one around 310°C, attributed to the reduction of free NiO particles, and a second one, very wide and centered around 550°C, assigned to the reduction of Ni species strongly interacting with the support. As commented above, the reduction of nickel aluminate occurred at temperatures greater than 800°C. For the Rh-Ni/CeO_2_ sample, the profile shows three different peaks: the lowest temperature peak, around 125°C, is assigned to the reduction of free Rh_2_O_3_ NPs. As the area of this peak is much higher than that corresponding to the total reduction of Rh in the sample, the consumption of hydrogen by ceria must be considered (Li et al., 2016).

The second one would be an overlapping peak comprising the reduction of Rh species strongly interacting with the support and the reduction of free NiO particles, while the third one, in the range of 650–800 °C, would be due to the reduction of complex NiOx species strongly interacting with the support, as well as surface ceria reduction. Finally, at temperatures higher than 800°C, the TCD signal is attributed to the reduction of bulk CeO_2_ species. Comparison of the reduction profiles of Rh-Ni/CeO_2_ and Ni/CeO_2_ indicates that the presence of Rh promotes CeO_2_ reduction, since the reduction profile of the Rh-Ni sample is shifted to lower temperatures, which can be attributed to hydrogen spillover during RhO_x_ reduction (Ocsachoque et al., 2016).

The reduction profile of the lanthana-supported Rh-Ni also showed two components—similar to what occurred in the Rh-free counterpart—due to the reduction of LaNiO_3_ which is known to occur in two stages. As commented above, the first was due to the reduction of Ni^3+^ to Ni^2+^, which is shifted to lower temperatures in comparison to Ni/La_2_O_3_, and the second saw LaNiO_2.5_ be reduced to finely dispersed metallic nickel NPs supported on La_2_O_3_.

The TPR profile of Rh-Ni/MgO is characterized by very low H_2_ consumption, as was the case for the Ni/MgO, which is due to the formation of Ni-Mg spinel in a larger extent during the calcination step. The small contribution found that around 300°C is attributed to the reduction of free NiO species. A small and wide peak was observed in the range of 400–750°C, which is associated to the reduction of Ni^2+^ interacting with the MgO lattice in external and subsurface layers. H_2_ consumption at temperatures higher than 750–800°C is likely associated to the reduction of Ni^2+^ in NiO-MgO solid-solution (Wang et al., 2009a). The reduction profile of Rh-Ni/ZrO_2_ shows three contributions: the first one, centered around 275°C, is assigned to the reduction of Rh oxide interacting with the support and also to free NiO particles; the second one, around 400°C, is due to surface nickel species interacting with the support, whereas the high temperature and wide reduction peak is due to the reduction of Ni^2+^ species inserted into the bulk zirconia lattice (Singha et al., 2016).

The interaction of nickel species with the support is one of the factors that influence the reactivity of the catalysts. In principle, a strong interaction among both phases is beneficial to stabilize the supported metal NPs, decreasing the deactivation by sintering (Ruckenstein and Wang, 1999).

#### Surface Composition

The binding energies of the Ni 2p, Al 2p, Ce 3d_5/2_, La 3d_5/2_, Mg 2p, and Zr 3d_5/2_ core levels, surface atomic ratios of Ni/(support cation) and metallic Ni and nickel oxide proportions have been determined by XPS. For the samples supported on ceria and lanthana, the analysis of the Ni 2p_3/2_ level is difficult since this level overlaps with the La 3d_3/2_ and Ce 3d_5/2_ levels and the quantification was done after careful deconvolution of both spectra.

**Figure 5:**
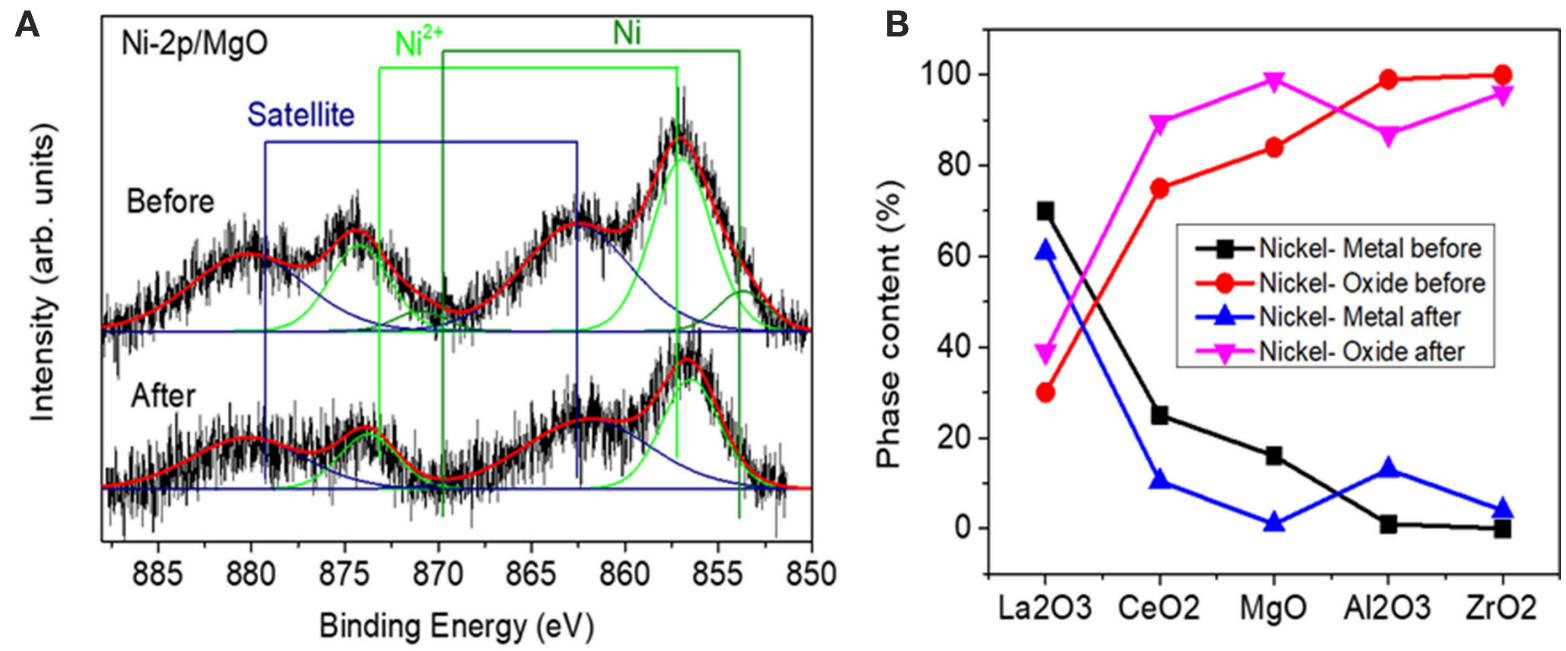
**(A)** Example of a fitted spectrum for the Ni/MgO sample **(B)** Relative content of metallic nickel and nickel oxide species extracted from the analysis of Ni-2p XPS data measured before and after reaction.

The Ni 2p XPS core level region was fitted with three doublets assigned to metallic Ni (2p3/2, 852.5 eV), Ni^2+^ (2p3/2,855–856.2 eV), and satellite features (861, 878.2 eV). An example of a fitted spectrum for the Ni/MgO sample is shown in Figure 5A. The percentage of phase content for each metallic and metal oxide species was calculated by integrating the fitted XPS data of each sample before and after the reaction. The evolution of the Ni oxidation state is shown in Figure 5B. Thus, for the calcined Ni samples, the concentration of surface metallic nickel shows the following trend: La_2_O_3_ > CeO_2_ > MgO > Al_2_O_3_ > ZrO_2_. The highest metallic nickel exposure was extracted for the sample supported on lanthana, related to the formation of LaNiO_3_, in which nickel is atomically distributed in the perovskite lattice. In the other extreme is the sample supported on zirconia, with the lowest metallic nickel proportion. For this series, the trend found for the surface Ni/substrate ratio is the following: La_2_O_3_ > MgO > ZrO_2_ > CeO_2_ > Al_2_O_3_. The highest value found for the catalyst supported on lanthana is explained by the atomic nickel dispersion achieved in LaNiO_3_ perovskite. The others follow the same trend of the surface areas of the substrates, with lower dispersion being found with a smaller surface area of the support, with the exception of the Ni/Al_2_O_3_ sample, for which the formation of some proportion of nickel aluminate is not discarded.

For the calcined Rh-Ni samples, the binding energy found for the Ni 2p_3/2_ level, around 856 eV and accompanied by a satellite peak around 6 eV higher, is characteristic of Ni^2+^ species. Rh was not analyzed since its low amount makes it difficult to be detected. The binding energies found for the support cation indicate that the surface support phases are as follows: Al_2_O_3_, CeO_2_, La_2_O_3_, MgO, and ZrO_2_. The C 1s spectra show a peak around 290 eV, corresponding to surface carbonates for the lanthana and magnesia-supported catalyst precursor, being much intense for the lanthana-supported one. The Ni/M (M: Al, Ce, La, Mg, Zr) surface ratios are compiled in Table 4. These ratios change considerably depending on the support type. The trend found is CeO_2_ > La_2_O_3_ > ZrO_2_ > Al_2_O_3_ > MgO. The higher ratios obtained for ceria and lanthana-supported catalyst precursors are characteristic of a highly dispersed nickel phase. The low Ni/Mg ratio is due to the formation of a Ni-Mg-O solid solution (Arena et al., 1996; Barbero et al., 2003). The C 1s core-level spectrum of the lanthana-supported catalyst precursor shows a component around 289–290 eV due to the presence of carbonates, which is in accordance with the strong basic character of this oxide.

**Table 4:** Binding energies (eV) and atomic surface ratios of calcined RhNi samples (M: Al, Ce, La, Mg, Zr) prepared by solid state reaction.

**Sample**	**Ni2p_**3/2**_**	**M2p(3d)**	**Ni/M at**	**${\text{CO}}_{3}^{2-}$/M at**
RhNi/Al_2_O_3_	856.0	74.5	0.044	–
RhNi/CeO_2_	856.2	883.1	0.497	–
RhNi/La_2_O_3_	855.6	834.7	0.389	1.303
RhNi/MgO	855.8	50.2	0.013	0.191
RhNi/ZrO_2_	855.5	182.2	0.263	–

### Physicochemical Characterization of Used Catalysts

#### Structural Properties

Rh-Ni catalysts after reaction have been structurally characterized by X-ray diffraction. X-ray diffraction patterns, depicted in Figure 1, showed that the reaction lead to structural changes in all the catalysts, as observed by comparing with the XRD patterns of fresh reduced samples. An increase in crystalline domain size of the support can be observed for the catalysts Rh-Ni/CeO_2_, Rh-Ni-MgO, and Rh-Ni/ZrO_2_ (Table 2). For the catalyst supported on lanthana, the support is based on lanthanum oxycarbonate (Requies et al., 2005; Navarro et al., 2007a), being formed by a reaction between La_2_O_3_ and CO_2_. Except for the RhNi/MgO catalyst, the other diffractograms show lines corresponding to metallic nickel, with its crystalline domain size being higher for the catalyst supported on ceria and smaller for the one supported on alumina. On the other hand, for the catalyst supported on ceria, diffraction lines corresponding to the formation of oxygen defective ceria (CeO_2−x_) can be observed (peak around 26.2°, ascribed to the diffraction plane (2 2 2), PDF card 00-049-1415).

#### Carbon Formation

**Figure 6:**
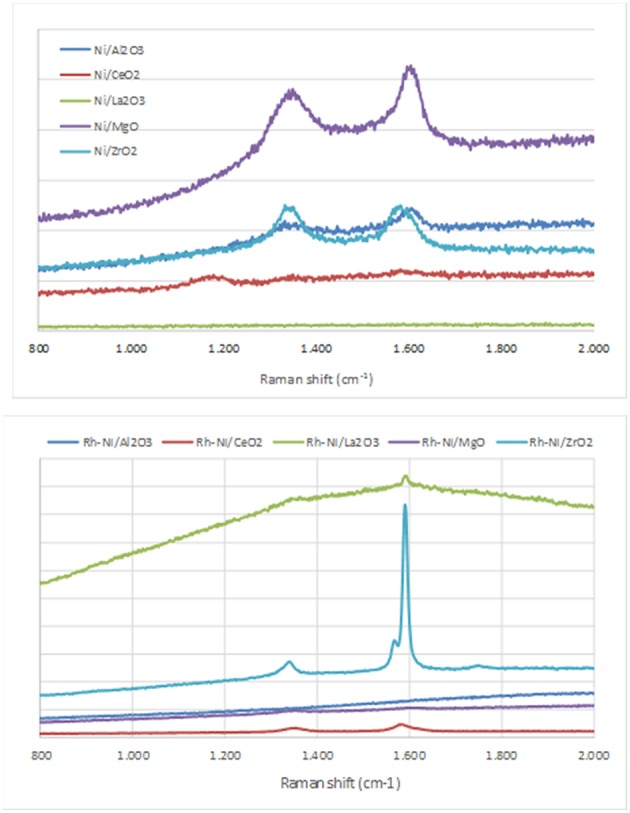
Raman spectra of the catalysts after reaction (Ni catalysts, prepared by impregnation and Rh-Ni catalysts prepared by solid-state reaction).

For CPOM, carbon deposition can take place by the Boudouard reaction (2 CO Δ C + CO_2_) and by the direct decomposition of methane (CH_4_ Δ C + 2 H_2_), (Pena et al., 1996). The characterization of carbon deposits on the spent catalysts was carried out by Raman spectroscopy (spectra are shown in Figure 6). For each sample, at least three Raman spectra were recorded in different areas to assure the homogeneity of the composition.

Raman spectra of carbon exhibited two quite sharp modes, the G (graphitic) peak around 1590 cm^−1^ and the D (disordered) peak around 1350 cm^−1^, which is related to the formation of carbon nanoparticles, amorphous carbon, or defective carbon filaments (Ozdemir et al., 2010; Lopez-Fonseca et al., 2012). The ceria-supported catalysts showed low intense peaks of carbon deposits. This is due to the widely reported oxygen mobility of the ceria surface (Dong et al., 2002). The low carbon formation in lanthana-supported catalysts is ascribed to the surface La_2_O_2_CO_3_ species, well known as gasifier agents of C precursors.

As observed in Figure 6, in the samples in which peaks corresponding to carbon deposits are found, with the exception of Ni/ZrO_2_, the graphitic band is higher than that of the disorder carbon one, particularly in the catalyst Rh-Ni/ZrO_2_ (Song et al., 2008). This spent catalyst presents an additional band around 1570 cm^−1^ as a consequence of the splitting of the G band, which is characteristic of carbon nanotubes (Kogler et al., 2014). The formation of different types of carbon seems to be related to the crystal phase of zirconia. As reported in the literature, a monoclinic phase favors the formation of encapsulating carbon and, for the tetragonal phase, the formation of carbon nanotubes is more likely (Zhang et al., 2015).

#### Surface Composition

**Figure 7:**
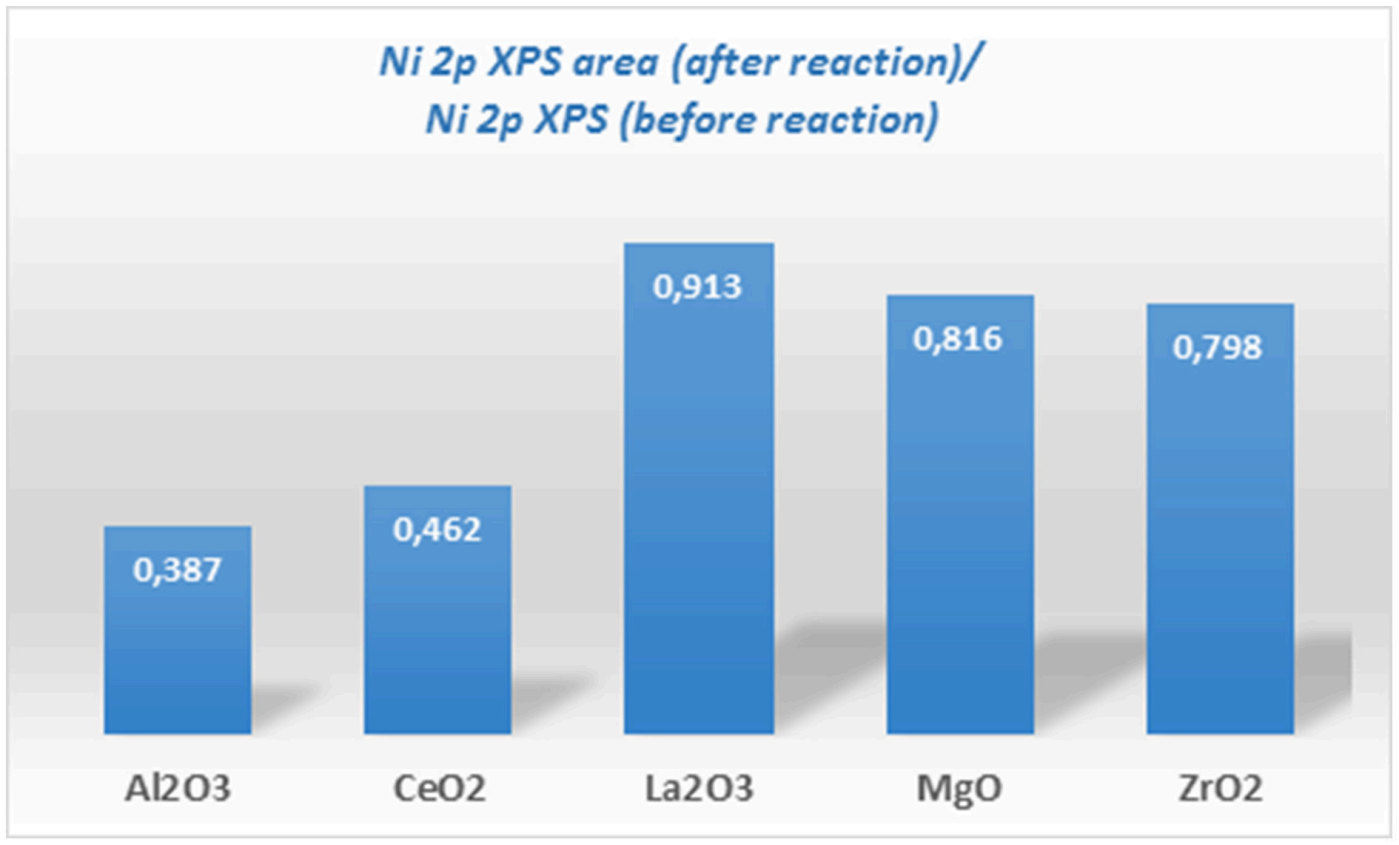
Ratio of Ni to substrate before and after the reaction of Ni catalysts supported on different substrates (prepared by impregnation) extracted from the analysis of XPS data.

Ni-supported catalysts were analyzed after reaction by XPS. Figure 5 shows the content of metallic Ni (%) and the nickel oxide (%) for each one. If these values are compared to those obtained for the catalysts before reaction (fresh calcined), a change of the surface metallic nickel exposure concentration can be observed depending on the catalyst type. Thus, for the samples supported on alumina and zirconia, an increase in the surface metallic nickel % is obtained, contrary to our observation for the other samples. On the other hand, in order to compare the evolution of the surface composition during reaction, another parameter has been compared for all the catalysts, which is depicted in Figure 7 and represents the ratio between the total Ni 2p area in the catalyst after and before reaction. These results point out that the total surface Ni area of the Ni catalyst supported on lanthana does not evolve significantly during reaction; on the contrary, the catalyst supported on alumina undergoes the greatest decrease in total surface nickel concentration.

### Activity Tests

**Figure 8:**
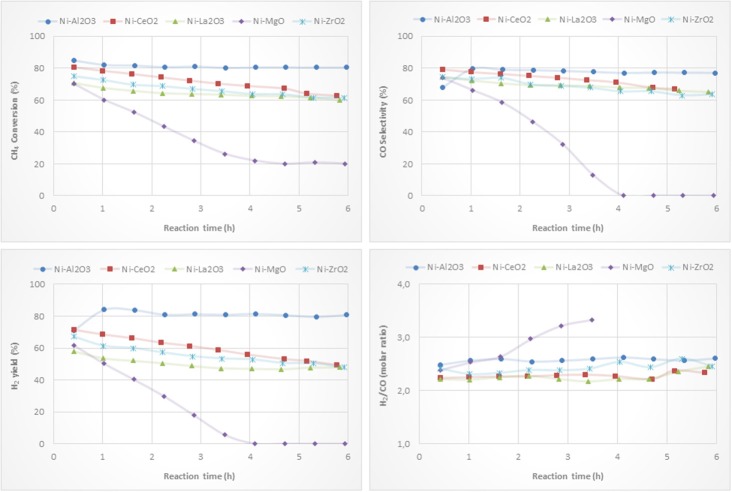
Catalytic performance of Ni catalysts (prepared by impregnation) for CPOM: CH_4_ conversion, H_2_ yield, CO selectivity and H_2_/CO molar ratio.

Ni-based catalysts prepared by impregnation were tested for the partial oxidation of methane to syngas and/or hydrogen. CH_4_ conversion, H_2_ yield, CO selectivity and H_2_/CO molar ratio versus reaction time are depicted in Figure 8. The catalytic behavior highly depends on the type of support. The most active, selective and stable catalyst is the one on alumina. The counterpart supported on magnesia, which suffers a drastic deactivation, shows the worst catalytic behavior. The beginning of the reaction depends on the catalyst support. The reactivity trends observed with the other catalysts are as follows: Ni/CeO_2_ > Ni/ZrO_2_ > Ni/La_2_O_3_. However, these catalysts suffer a different deactivation rate, but similar values for methane conversion, H_2_ yield and CO selectivity were found after 6 h of reaction. The better performance showed by the Ni/Al_2_O_3_ catalyst is mainly attributed to a relatively high level of nickel dispersion, favored by the higher surface area of the alumina support.

Although the metal-to-oxide ratio for Ni/La_2_O_3_ (before and after reaction) (Figure 5) is much higher than the analogous ratios of the other catalysts, and the Ni 2p area barely decreases during reaction (Figure 7), in agreement with earlier findings (Requies et al., 2005), this catalyst does not appear to be very active for CPOM. Since the analysis of the Raman spectra rules out the carbon formation and therefore carbon deposition on nickel species, the observed deactivation should be due to sintering and/or the coverage of nickel species by lanthana carbonates, which are produced by the adsorption of CO_2_ formed during the reaction (Requies et al., 2005). The deactivation of this catalyst is explained by its metal/support ratio (Figure 5), far from the optimum to favor the selectivity to methane partial oxidation, for this type of support. Furthermore, the oxidation of nickel particles at the beginning of the reaction would lead to lower selectivity toward syngas or hydrogen formation, increasing the selectivity toward total oxidation and, consequently, catalyst deactivation.

As reported in the literature, the catalytic performance of a catalyst based on Ni/MgO depends on its composition, preparation conditions, and even the properties of the MgO support (Hu and Ruckenstein, 2004; Nguyen et al., 2016). The fast deactivation observed for the Ni/MgO catalyst is due to the low fraction of metallic Ni on the catalyst surface (~7%). Indeed, this Ni-loading is far below the optimal range (10-35%). As a consequence, this catalyst shows very low activity for CPOM (Ruckenstein and Hu, 1999), with only CO_2_ and carbon as products after 4 h on-stream. Moreover, the nickel fouling leads to a substantial decrease in the Ni-support ratio (Figure 5). The deactivation of the Ni/ZrO_2_ catalyst is mainly related to the formation of carbon deposits (Figure 6), which agrees with the results reported in the literature (Pengpanich et al., 2004; Larimi and Alavi, 2012).

The H_2_/CO ratios are higher than 2 and lower than 2.7, except for the catalyst supported on magnesia, because this catalyst behaves as a combustion catalyst. For the other catalysts, the trend found for the H_2_/CO ratio mainly depends on the extent of the Boudouard reaction (2 CO Δ CO_2_ + C) and reverse water gas shift reaction, rWGS (CO_2_ + H_2_ Δ CO + H_2_O), with this ratio being higher when the Boudouard reaction occurs at a large extent and the rWGS reaction at a low extent (Albarazi et al., 2013). Thus, it appears that a direct correlation between the Raman peaks related to carbon formation (Figure 6) and the H_2_/CO ratio can be observed.

**Figure 9:**
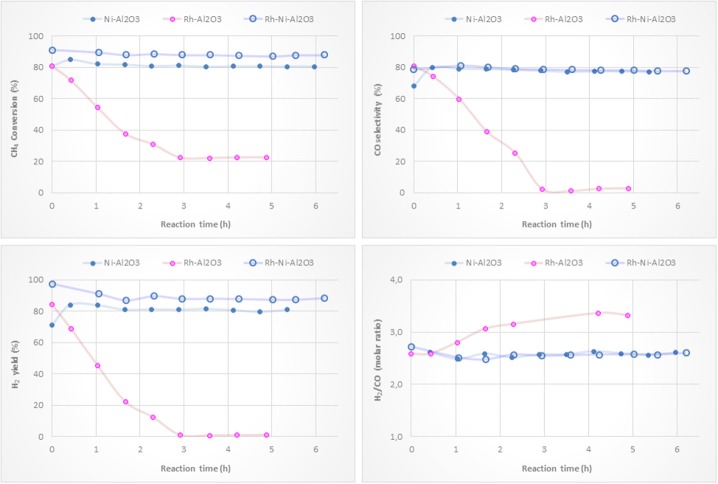
Catalytic performance of Ni/Al_2_O_3_, Rh/Al_2_O_3_ and RhNi/Al_2_O_3_ catalysts (prepared by impregnation) for CPOM: CH_4_ conversion, H_2_ yield, CO selectivity and H_2_/CO molar ratio.

Figure 9 shows activity tests with monometallic Ni and Rh catalysts supported on alumina and with the bimetallic counterpart. Methane conversions and H_2_ yields clearly show that adding a small amount of Rh to the nickel catalyst results in an improvement in the catalytic performance of the Ni-based catalyst. This is reasonably explained by the higher reduction degree of surface nickel particles by the H_2_ spillover originating on Rh NPs (see Figure 4). The Rh monometallic catalyst suffers from fast deactivation produced as a consequence of the low amount of active phase (Hohn and Schmidt, 2001; Berger-Karin et al., 2011).

**Figure 10:**
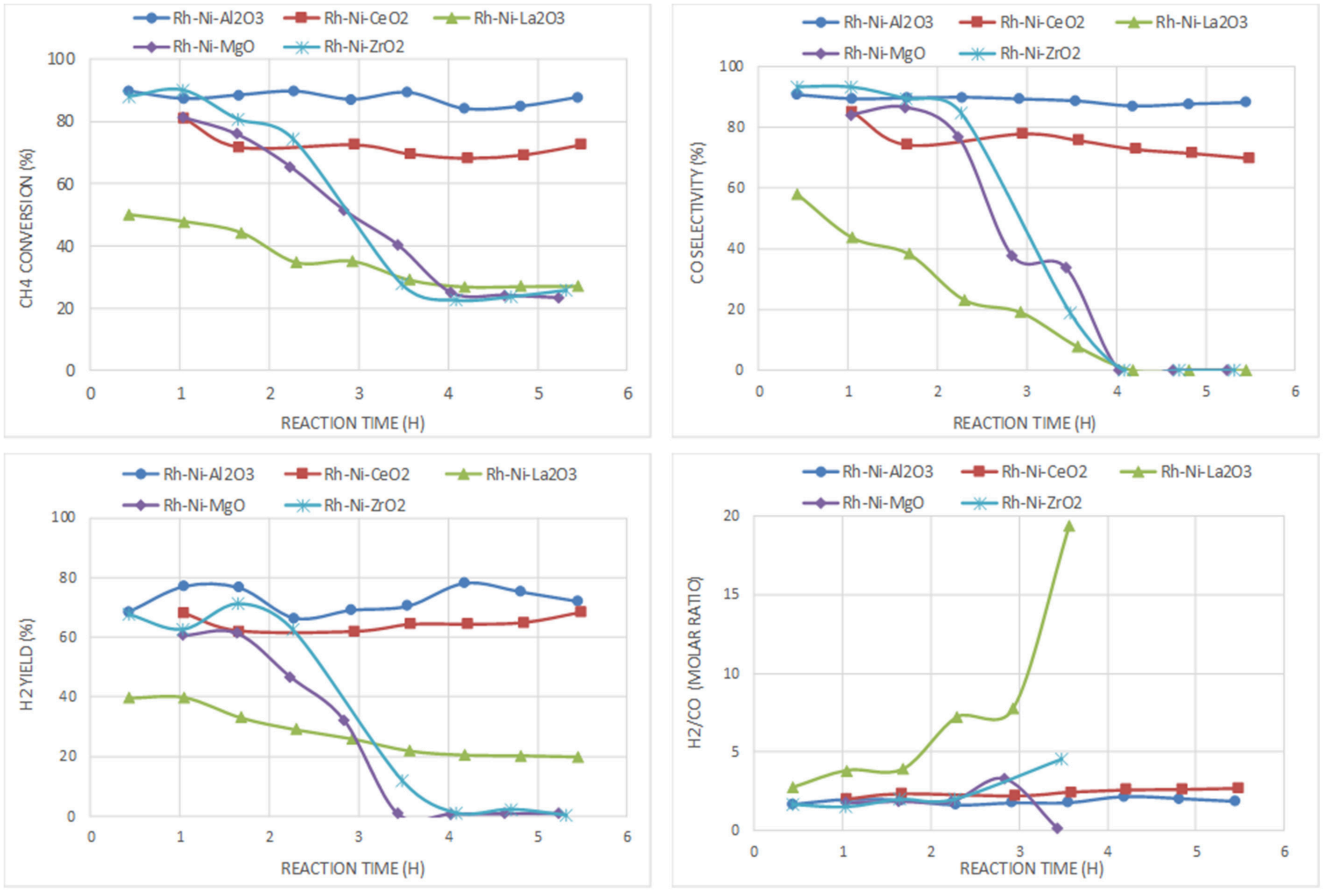
Catalytic performance of Rh-Ni catalysts (prepared by solid state reaction) for CPOM: CH_4_ conversion, H_2_ yield, CO selectivity and H_2_/CO molar ratio.

For the catalyst series prepared by the solid-state reaction based on Rh and Ni as active phases (Figure 10), the best performance is also obtained with the catalyst supported on alumina. The ceria-supported catalyst showed also good performance with a H_2_ yield similar after 6 h on stream, even having a lower surface area (Table 3). The low deactivation of the RhNi/CeO_2_ catalyst by carbon formation is a consequence of the oxygen mobility and storage of the ceria support promoted by the redox pair Ce(IV)/Ce(III) (Pantaleo et al., 2016), which is known to play a key role in this reaction (Ding et al., 2015). The results obtained by O_2_ pulses at reaction temperature point out that the extent of O_2_ chemisorbed in this catalyst is 1.344 mmol/g, a much higher amount than on the other catalysts (RhNi/Al_2_O_3_: 0.127; RhNi/La_2_O_3_: 0.144; RhNi/MgO: 0.299; RhNi/ZrO_2_: 0.546 mmol/g).

The other catalysts undergo fast deactivation with time on-stream. The deactivation of the catalyst supported on zirconia is explained by the fouling of active phases, since they underwent extensive carbon formation. The deactivation rate of the zirconia-supported bimetallic catalysts is higher than that observed for the monometallic counterpart. The different crystalline phases of zirconia (monoclinic for the monometallic catalyst, prepared by impregnation, and tetragonal, for the bimetallic one, prepared by solid state reaction), may have an influence on carbon formation. As reported in the literature, the ability of CO_2_ activation, which is essential to remove the surface carbon species, is related to the crystal phase of ZrO_2_ since different active sites are expected to be present on the different surfaces (Zhang et al., 2015). Thus, it has been reported that CO_2_ reacts by the reverse Boudouard reaction, with carbon species resulting from methane dissociation to CO (Baerns et al., 1997). The higher CO_2_ adsorption capacity of m-ZrO_2_ is attributed to the higher concentration and basicity of the hydroxyl groups on this polymorph, as well as to the stronger Lewis basicity of O_2_-anions (Pokrovski et al., 2001). This fast deactivation during CPOM has also been reported in a study using a monoclinic zirconia-supported Ni catalyst, also prepared by solid state reaction (Choudhary et al., 1998b).

Another factor influencing the reactivity of this catalyst is the sintering of nickel produced by hot spot formation in the catalyst bed, because the space velocity may not be high enough (Tang et al., 1998; Hohn and Schmidt, 2001). Since the interaction of metallic nickel particles on ZrO_2_ is relatively weak, the catalysts are prone to deactivate by sintering of nickel particles (Barbero et al., 2003). Such species can be observed in the XRD diffraction lines of the spent catalyst (Table 2).

For the catalyst supported on magnesia, in which mainly an unreduced Ni-Mg-O solid solution was found that was inactive for the CPOM reaction, the decrease in selectivity toward syngas or H_2_ was influenced by the low surface proportion of metallic nickel (Requies et al., 2005). Therefore, this catalyst deactivated gradually due to its oxidation (Nurunnabi et al., 2006). Although this mixed oxide phase inhibits carbon deposition, especially from CO disproportionation (Tang et al., 1998), the surface content of nickel produced from Ni-Mg-O reduction is too low (or the MgO surface proportion too large). The analysis by Raman spectroscopy of this catalyst after reaction revealed the near absence of carbon, which runs contrary to the result obtained for the monometallic catalyst (Ni/MgO). This is explained by the role of Rh, which enhances the resistance to carbon deposition (Nurunnabi et al., 2006).

In the case of the catalyst supported on lanthana, it displays the worst catalytic performance, with an initial methane conversion of 50%, which is almost half the conversion achieved by the other catalysts. Its deactivation is not due to an extensive carbon formation, since only a small peak around 1,600 cm^−1^, attributed to graphitic carbon, is observed (Figure 6). In this catalyst, carbon is gasified by lanthanum oxycarbonate species (Figure 1) that limits deactivation to some extent.

In summary, the most active and selective catalysts are those supported on alumina and ceria. The comparison between Ni/Al_2_O_3_ and RhNi/Al_2_O_3_, as well as between Ni/CeO_2_ and RhNi/CeO_2_, points out that the bimetallic ones are more active, selective and stable. This is influenced by the greater metal-support interface achieved in these catalysts, prepared by solid state reaction, by the higher intrinsic activity of Rh and its role favoring the reduction of Ni.

The catalysts that have showed better activity per surface area for CPOM under the reaction conditions used in this research are those supported on ceria (Ni/CeO_2_ and RhNi/CeO_2_). In these catalysts, methane is dissociated on nickel particles, and C species migrate to the interface with the support to form CO (Dong et al., 2002). The bimetallic catalyst showed higher methane conversion, higher selectivity to H_2_ and syngas and higher stability as a consequence of the larger metal-support interface. One of the properties that improved the catalytic behavior of this system is the non-stoichiometry of ceria, in which there is more oxygen mobility as compared to the other supports, which plays a key role in oxygen adsorption and carbon gasification (Pengpanich et al., 2004).

## Conclusions

Catalysts based on Ni, Rh, and Rh-Ni supported on Al_2_O_3_, CeO_2_, La_2_O_3_, MgO, and ZrO_2_ have been prepared by wet impregnation of commercial supports and by solid state reaction. The catalysts have been tested for CPOM and it was found that their performance depends on the support type and on the preparation method. It can also be observed that a small amount of Rh promotes the reduction of Ni species, improving the catalytic performance. Regardless of the preparation method, the most active, selective and stable catalysts are those supported on alumina, which is mainly due to the higher dispersion of the nickel particles. The Ni/CeO_2_ catalyst shows the best behavior per surface area, in which the lack of stoichiometry of the CeO_2_ is a key property influencing the reactivity and stability. The catalyst composition of RhNi-CeO_2_ prepared by solid state reaction yields higher activity and stability than that of Ni-CeO_2_, prepared by impregnation. The fast deactivation of the catalysts supported on magnesia is explained by the low amount of active Ni sites, which leads to the combustion of methane, rather than to its selective oxidation. For the catalysts supported on lanthana, the deactivation is ascribed to the nickel content being lower than the optimum required amount on this type of support, resulting in the oxidation of nickel. The deactivation of the zirconia-supported catalysts is ascribed to the extensive formation of carbon, being less pronounced in the Ni/ZrO_2_ catalyst due to the higher basicity of monoclinic zirconia in comparison to the tetragonal phase of RhNi/ZrO_2_.

## Data Availability

All datasets generated for this study are included in the manuscript and/or the supplementary files.

## Author Contributions

CA-G, MM, LR-M, and JE contributed to the preparation, characterization of catalysts and catalytic activity tests. RN contributed to tune up the reaction system and GC. MA, BR, and JF discussed the XPS results. CA-G discussed the results obtained by the other characterization techniques and the catalytic activity tests data.

### Conflict of Interest Statement

The authors declare that the research was conducted in the absence of any commercial or financial relationships that could be construed as a potential conflict of interest.
